# Supplementary material for: From Mutation to Prognosis: AI-HOPE-PI3K Enables Artificial Intelligence Agent-Driven Integration of PI3K Pathway Data in Colorectal Cancer Precision Medicine
Source: Int J Mol Sci. 2025 Jul 5;26(13):6487. doi: 10.3390/ijms26136487 (PMC12249626; doi:10.3390/ijms26136487)
Supplement: Supplementary file 1 [file ijms-26-06487-s001.zip › ijms-3692214-supplementary.pdf]

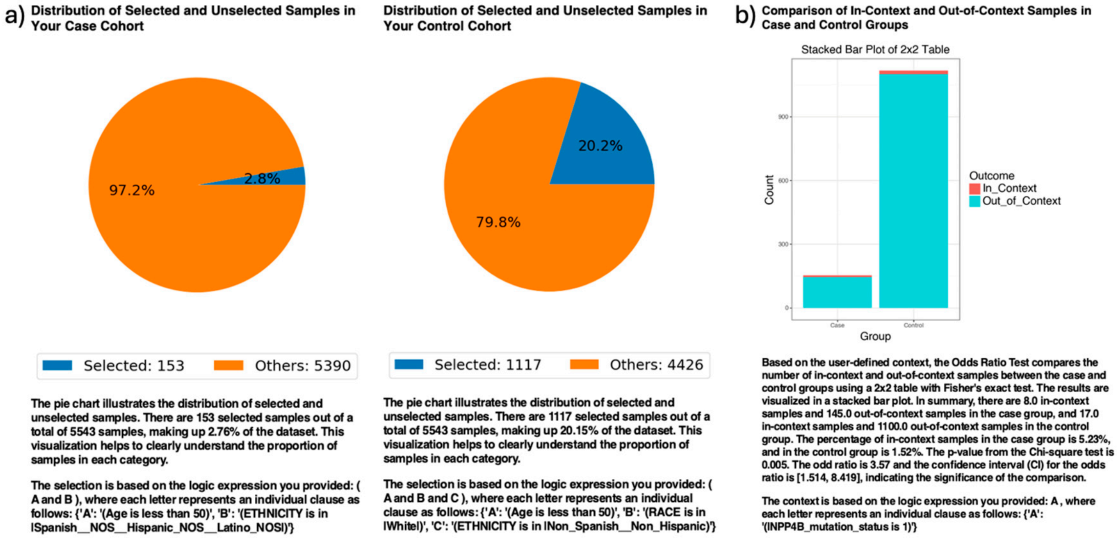

Figure S1. AI-HOPE-PI3K analysis of INPP4B mutations in early-onset colorectal cancer (EOCRC) among Hispanic/Latino (H/L) and Non-Hispanic White (NHW) patients. a) Pie charts illustrate the distribution of selected samples following natural language–driven filtering of the dataset. The case cohort includes 153 EOCRC patients under age 50 with H/L ethnicity, representing 2.76% of the dataset. The control cohort includes 1,117 EOCRC patients under age 50 who are NHW, representing 20.15% of the dataset. b) A 2×2 odds ratio analysis evaluates the frequency of INPP4B mutations between the two groups. The stacked bar plot shows the proportion of samples with and without INPP4B mutations, labeled as "In_Context" and "Out_of_Context," respectively. INPP4B mutations were present in 5.23% of H/L cases and 1.52% of NHW controls. The calculated odds ratio was 3.57 (95% CI: 1.514–8.419), with a p-value of 0.005, indicating a statistically significant difference.

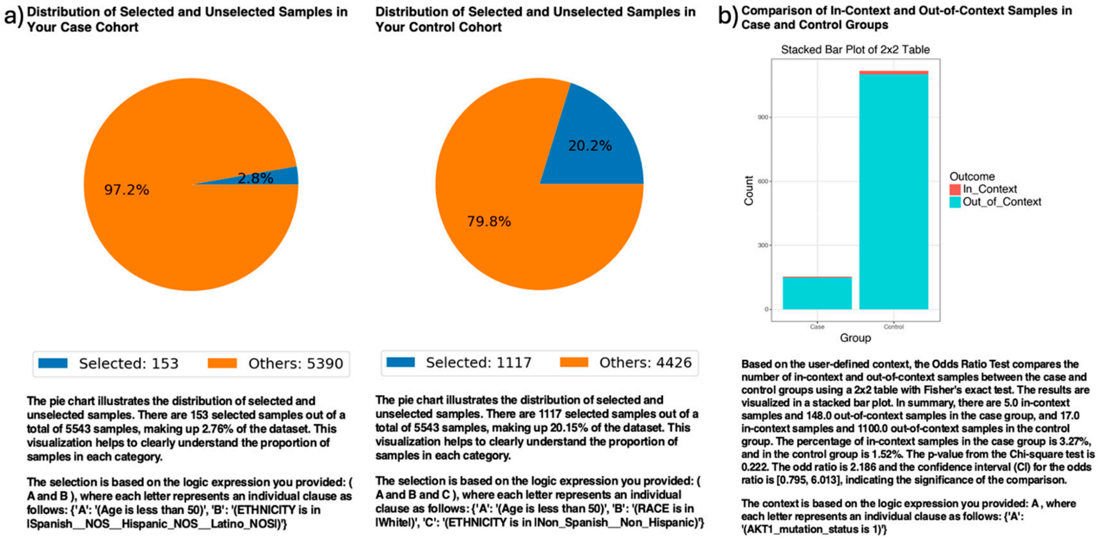

Figure S2. AI-HOPE-PI3K analysis of AKT1 mutations in early-onset colorectal cancer (EOCRC) among Hispanic/Latino (H/L) and Non-Hispanic White (NHW) patients. a) Pie charts illustrate the distribution of selected samples following natural language–driven filtering of the dataset. The case cohort includes 153 EOCRC patients under age 50 with H/L ethnicity, representing 2.76% of the dataset. The control cohort includes 1,117 EOCRC

patients under age 50 who are NHW, representing 20.15% of the dataset. b) A 2×2 odds ratio analysis evaluates the frequency of AKT1 mutations between the two groups. The stacked bar plot shows the proportion of samples with and without AKT1 mutations, labeled as "In_Context" and "Out_of_Context," respectively. AKT1 mutations were present in 3.27% of H/L cases and 1.52% of NHW controls. The calculated odds ratio was 2.19 (95% CI: 0.795–6.013), with a p-value of 0.222, indicating no statistically significant difference.

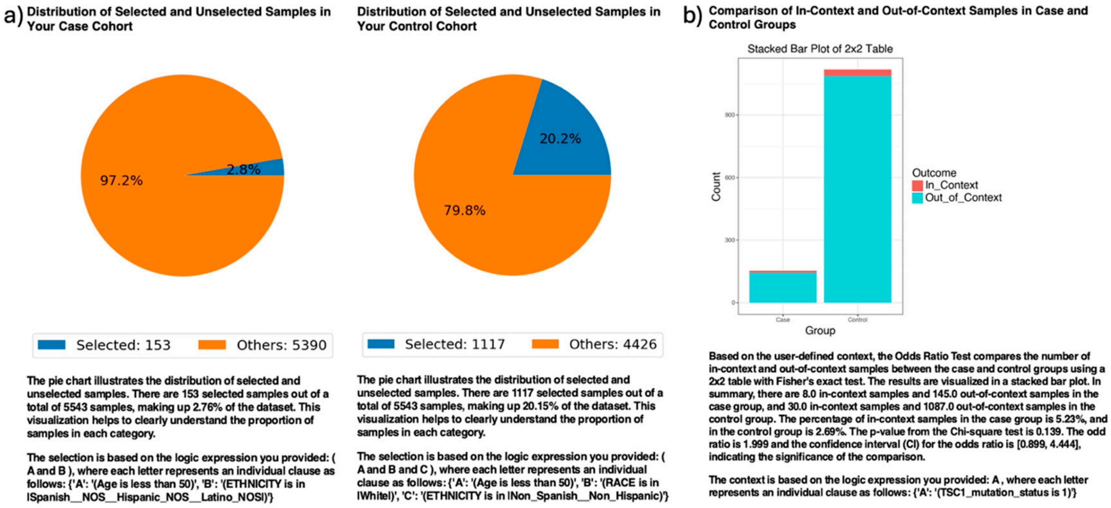

Figure S3. AI-HOPE-PI3K analysis of TSC1 mutations in early-onset colorectal cancer (EOCRC) among Hispanic/Latino (H/L) and Non-Hispanic White (NHW) patients. a) Pie charts illustrate the distribution of selected samples following natural language–driven filtering of the dataset. The case cohort includes 153 EOCRC patients under age 50 with H/L ethnicity, representing 2.76% of the dataset. The control cohort includes 1,117 EOCRC patients under age 50 who are NHW, representing 20.15% of the dataset. b) A 2×2 odds ratio analysis evaluates the frequency of TSC1 mutations between the two groups. The stacked bar plot displays the proportion of samples with and without TSC1 mutations, labeled as "In_Context" and "Out_of_Context," respectively. TSC1 mutations were present in 5.23% of H/L cases and 2.69% of NHW controls. The calculated odds ratio was 2.00 (95% CI: 0.899–4.444), with a p-value of 0.139, indicating no statistically significant difference.

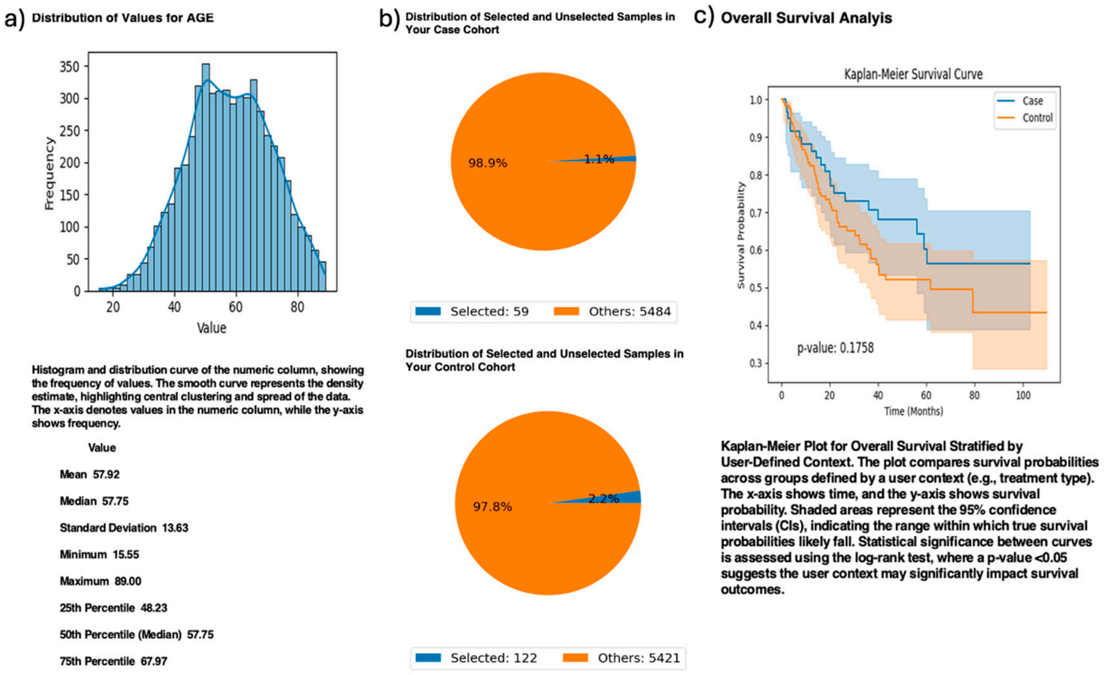

Figure S4. AI-HOPE-PI3K analysis of PTEN-mutated colorectal cancer (CRC) patients treated with FOLFOX chemotherapy, stratified by age group. This figure illustrates the application of AI-HOPE-PI3K to assess survival outcomes in PTEN-mutated CRC patients treated with the FOLFOX regimen (Fluorouracil, Leucovorin, Oxaliplatin), comparing early-onset (<50 years) and late-onset (>50 years) cases. a) A histogram depicts the distribution of patient age across the dataset, with a mean of 57.92 years and a median of 57.75. The smooth curve overlay shows central clustering and spread of the data, contextualizing the age cutoff used for cohort stratification. b) Pie charts illustrate the cohort selection process following natural language query execution. The case cohort includes 59 early-onset CRC patients with PTEN mutations treated with FOLFOX (1.1% of the dataset), while the control cohort includes 122 late-onset CRC patients with the same molecular and treatment profile (2.2%). c) Kaplan-Meier survival analysis compares overall survival between early-onset and late-onset groups. Although early-onset patients demonstrated a course toward improved survival, the difference was not statistically significant (p = 0.1758). Shaded regions represent the 95% confidence intervals, suggesting overlapping survival distributions and no conclusive evidence of age-based survival differences in this PTEN-mutated, FOLFOX-treated CRC subgroup.

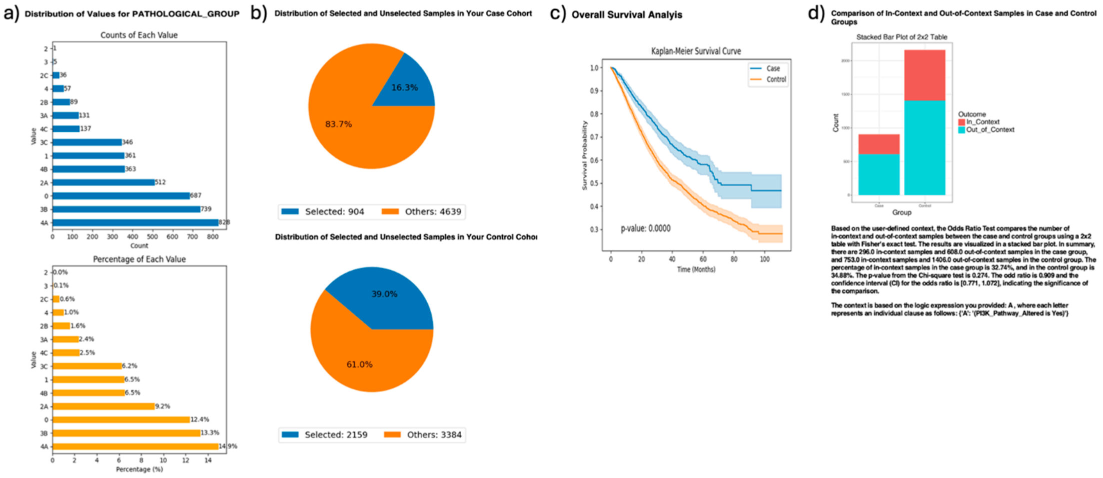

Figure S5. AI-HOPE-PI3K analysis of PI3K-altered colorectal cancer (CRC) stratified by pathological stage group: Early (Stage 0–2C) vs. Advanced (Stage 3–4C) disease. This figure demonstrates the application of AI-HOPE-PI3K to evaluate survival outcomes and molecular enrichment among CRC patients harboring PI3K pathway alterations, stratified by pathological stage at diagnosis. All patients received FOLFOX chemotherapy (Fluorouracil, Leucovorin, Oxaliplatin). a) Bar plots depict the distribution of pathological stage groups across the dataset. Early-stage cases (Stage 0, 1, 2, 2A, 2B, 2C; labeled X0) represent the majority of samples (54.2%), while advanced-stage cases (Stage 3A–4C; labeled X1) account for 45.8%. The upper panel shows the sample count per stage category, and the lower panel presents their proportional representation. b) Pie charts visualize the cohort selection process following natural language–driven filtering. The case cohort includes 628 early-stage CRC patients with PI3K pathway alterations treated with FOLFOX (11.3% of the dataset), while the control cohort consists of 507 advanced-stage PI3K-altered CRC patients treated with the same regimen (9.1%). These distributions contextualize cohort sizes and selection logic. c) Kaplan-Meier survival curves compare overall survival between the two groups. Despite a visual course potentially suggesting improved survival in the early-stage PI3K-altered group, the difference was not statistically significant (p = 0.1267). Shaded regions represent 95% confidence intervals. d) A multivariate odds ratio analysis was conducted to assess the association between PI3K pathway alterations and pathological stage.
